# Microwave-induced thermoacoustic imaging for the early detection of canine intracerebral hemorrhage

**DOI:** 10.3389/fphys.2022.1067948

**Published:** 2022-11-16

**Authors:** Jiawu Li, Zhenru Wu, Chihan Peng, Ling Song, Yan Luo

**Affiliations:** ^1^ Department of Ultrasound, West China Hospital of Sichuan University, Chengdu, China; ^2^ Institute of Clinical Pathology, Key Laboratory of Transplant Engineering and Immunology, NHC, West China Hospital, Sichuan University, Chengdu, China

**Keywords:** microwave-induced thermoacoustic imaging, canine, intracerebral hemorrhage, early detection, thermoacoustic signal

## Abstract

**Purpose:** This study aimed to investigate the feasibility and validation of microwave-induced thermoacoustic imaging (TAI) for the early detection of canine intracerebral hemorrhage.

**Methods:** A TAI system was used to record the thermoacoustic signal (TAS) of canine intracerebral hemorrhage in the study. First, the difference in TAS between deionized water, fresh *ex vivo* porcine blood and brain tissue was explored. Second, the canine hemorrhagic stroke model was established, and canine brain ultrasound examination and TAI examination were performed before modeling and at 0.5 h, 1 h, 2 h, 3 h, 4 h, 4.5 h, 5 h and 6 h after modeling. Finally, pathology and ultrasound were used as the reference diagnoses to verify the accuracy of the thermoacoustic imaging data.

**Results:** The results showed that significant differences were observed in TASs among deionized water, fresh *ex vivo* porcine blood and brain tissue. The intensity of the thermoacoustic signal of blood was significantly higher than that of *ex vivo* porcine brain tissue and deionized water. The intracerebral hemorrhage model of five beagles was successfully established. Hematomas presented hyperintensity in TAI. Considering ultrasound and pathology as reference diagnoses, TAI can be used to visualize canine intracerebral hemorrhage at 0.5 h, 1 h, 2 h, 3 h, 4 h, 4.5 h, 5 h and 6 h after modeling.

**Conclusion:** This is the first experimental study to explore the use of TAI in the detection of intracerebral hemorrhage in large live animals (canine). The results indicated that TAI could detect canine intracerebral hemorrhage in the early stage and has the potential to be a rapid and noninvasive method for the detection of intracerebral hemorrhage in humans.

## Introduction

Stroke is the second leading cause of death and the third leading cause of disability worldwide, including hemorrhagic and ischemic strokes, and its incidence is increasing, especially in developing countries (Kyu et al., 2018; Feigin et al., 2021). Because the two types of stroke have different causes, their treatments are also completely distinct. Thrombolysis is the main treatment for ischemic stroke, and the “effective treatment window” is very short (within 4.5 h), while thrombolysis is strictly prohibited for hemorrhagic stroke (Powers et al., 2019; Berge et al., 2021). However, the proportion of patients undergoing thrombolytic therapy for acute ischemic stroke is still very low. The principal reason is that it is difficult to effectively distinguish ischemic stroke from hemorrhagic stroke within 3–4.5 h of onset, which leads to the inability to carry out timely thrombolytic therapy for patients with ischemic stroke (Wang et al., 2011; El Khoury et al., 2012). Therefore, it is important to quickly and accurately distinguish hemorrhagic stroke from ischemic stroke.

At present, CT scans are the initial choice for the imaging diagnosis of acute stroke. MRI is more sensitive than CT in the diagnosis of ischemic foci or hematoma within 6 h after the onset of stroke (Chalela et al., 2007), but it is not recommended to use MRI for the early diagnosis of suspected stroke (Powers et al., 2018). However, CT and MRI are difficult to use for detecting intracranial hemorrhage in prehospital conditions or even at the scene of onset due to the large equipment needed. Researchers have been pursuing a portable, prehospital accessible method to accurately detect intracerebral bleeding (Mobashsher et al., 2016; Candefjord et al., 2017; Ljungqvist et al., 2017) to reduce the waiting time for preoperative examination and improve the prognosis of patients with hemorrhagic stroke.

Previous studies have shown that there is an obvious contrast between the conductivity of blood and white matter and gray matter (Peyman et al., 2007), in which the conductivity of gray matter, white matter and blood at a frequency of 3.0 GHz are 2.2189 S/m, 1.5106 S/m and 3.0498 S/m, respectively. The conductivity of blood is significantly higher than that of white matter and gray matter. Of note, in recent years, some studies (Persson et al., 2014; Candefjord et al., 2017; Ljungqvist et al., 2017) have used microwave imaging (MI) based on conductivity to detect acute stroke, and the results showed that brain MI could distinguish hemorrhagic stroke from ischemic stroke. However, the spatial resolution of the MI system is poor. It is difficult to meet the clinical need because the size and location of intracerebral hematoma cannot be provided. Further improvement of the spatial resolution of microwave imaging may make the evaluation of intracerebral hematoma more accurate.

Microwave-induced thermoacoustic imaging (TAI) is a novel noninvasive imaging modality that receives ultrasonic signals generated by the absorption of microwaves in different biological tissues and reconstructs the image to reflect the dielectric properties of biological tissues (Huang et al., 2012; Zheng et al., 2018; Sun et al., 2021; Lin, 2022). It has the advantages of the high contrast of microwave imaging and the high resolution of ultrasound imaging. Currently, TAI technology has attracted increasing attention based on its unique advantages and researchers are actively developing its huge potential role in biomedical diagnosis and even treatment. Differences in conductivity and relative permittivity between different tissues or normal tissues and lesions allow TAI to distinguish them from each other. The image resolution of TAI is determined by the detected ultrasonic signal. Compared with MI, TAI has higher spatial resolution due to its resolution is sub-millimeter (Liu et al., 2022). Up to now, TAI studies in the biomedical field have mainly involved the detection of breast cancer and prostate cancer, joint related disease and brain imaging, etc., especially in the early detection of breast cancer has been extensively studied (Liu et al., 2018; Li et al., 2019; Yuan et al., 2019; Li et al., 2021; Zhang et al., 2022). To date, there are few studies on TAI in brain disease. Xu et al. (Yuan and Wang, 2006) applied TAI to imaging the rhesus monkey brain through the intact skull, and the results showed that the brain parenchyma could be clearly visualized. Huang et al. (2017) made the first preliminary attempt to detect the hemorrhage phantom with the self-built TAI system in the world and successfully imitated a simulated hemorrhage phantom beneath an isolated human skull. The study of Yan et al. (2019) also indicated that thermoacoustic tomography can take images through the adult human skull. Zhao et al. (2017) showed that thermoacoustic tomography could visualize numerous important brain anatomical structures in rats, and they further attempted to detect germinal matrix hemorrhage in neonatal mice (Zhao et al., 2020). The results showed that thermoacoustic tomography can accurately detect a hematoma region at different depths in the neonatal mouse brain. However, to our knowledge, there is no relevant report on the study of thermoacoustic imaging in detecting intracerebral hemorrhage in large animals or humans. Therefore, the main purpose of this study was to investigate the feasibility and validation of thermoacoustic imaging for the rapid detection of canine intracerebral hemorrhage.

## Methods

The animal study was reviewed and approved by the Animal Ethics Committee of West China Hospital of Sichuan University. All applicate regulations concerning the ethical use of animals were strictly followed during the whole experiment. In this study, we first explored whether there was a difference in thermoacoustic signals among deionized water, fresh *ex vivo* porcine blood and brain tissue. Second, a canine hemorrhagic stroke model was established, and canine brain ultrasound examination and TAI examination were performed before modeling and at 0.5 h, 1 h, 2 h, 3 h, 4 h, 4.5 h, 5 h and 6 h after modeling. Finally, the dogs were sacrificed by air embolization under anesthesia, and their brains were removed and prepared for pathological examination to further verify the accuracy of thermoacoustic imaging.

### Microwave-induced thermoacoustic imaging system

**FIGURE 1:**
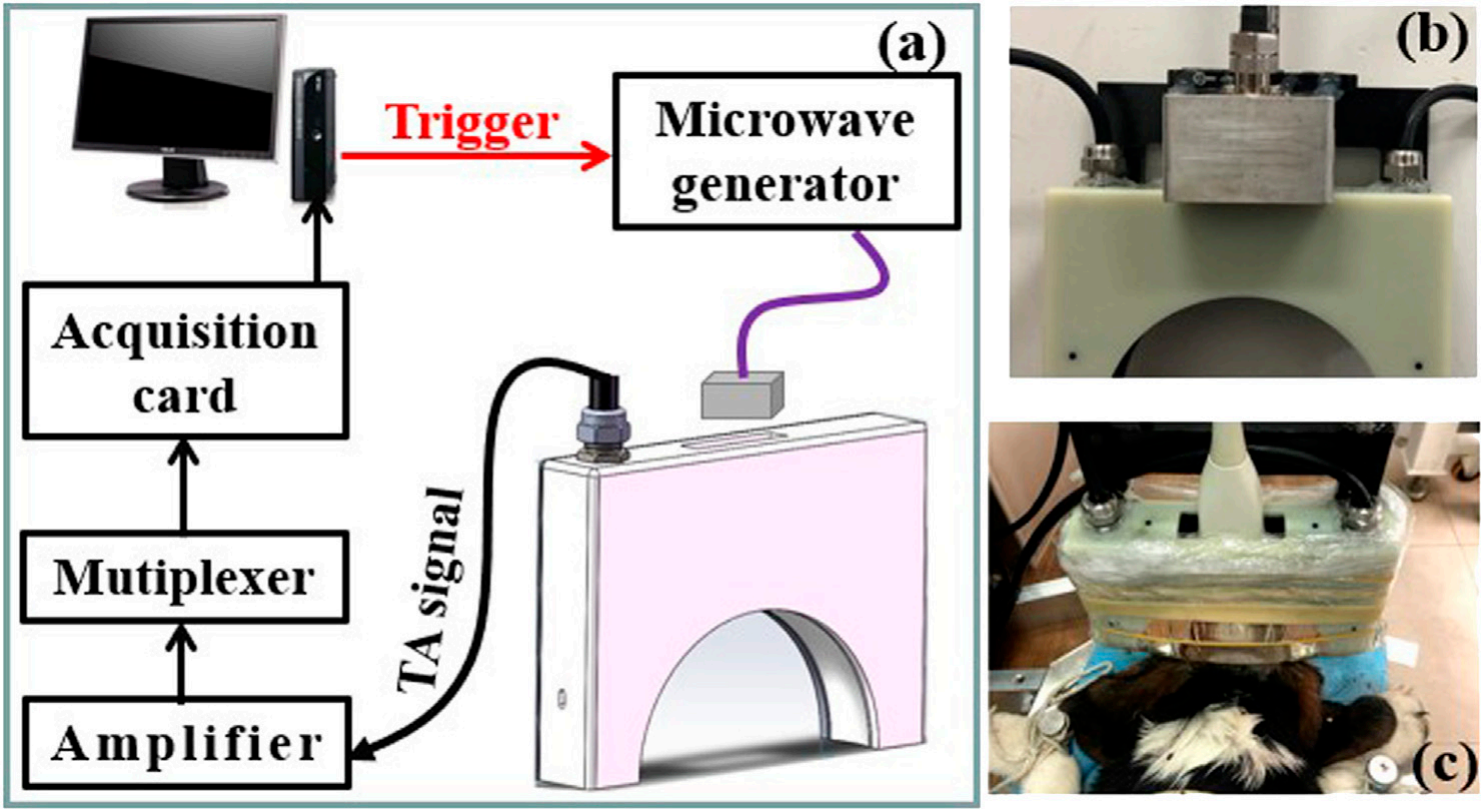
**(A)** Schematic of the thermoacoustic imaging (TAI) system. **(B,C)** Photograph of the antenna and hollow concave transducer array, the dog brain to be imaged, respectively.

A schematic of the TAI system utilized in this study is shown in Figure 1. A custom-designed miniaturized microwave generator (peak power: 60 kW, pulse width: 70–600 ns and repetition rate: 100 Hz) (Huang et al., 2021) coupled with a handheld dipole antenna (aperture size: 60 × 60 mm^2^) (Huang et al., 2019) *via* a semirigid coaxial cable (1.5 m long with 1.2 dB insertion loss) was used to evoke thermoacoustic signals. The TA signals were detected by a 128-element hollow concave transducer array (Wang et al., 2021), amplified and averaged 50 times to achieve a good signal-and-noise ratio, and finally recorded by a 64-channel acquisition system with two 32-channel data acquisition cards (5752B, NI. Inc., United States). A back-projection algorithm in MATLAB was used for TA image reconstruction (Hoelen and de Mul., 2000). A B-mode ultrasound imaging platform (iNSIGHT-37°C, SASET. Inc., China) was also used for imaging the dog brains.

### Thermoacoustic signal of deionized water, fresh *ex vivo* porcine blood and *ex vivo* porcine brain by thermoacoustic imaging

Considering the accessibility of *ex vivo* animal experimental materials, porcine blood and brain tissue were used in this part of the *in vitro* experiment. Fresh *ex vivo* porcine blood and brain tissue were obtained from a local slaughterhouse and were wrapped in aluminum foil to keep them fresh before experiments. In the experiment, deionized water, *ex vivo* porcine blood and brain tissue were placed into transparent plastic tubes with a diameter of 7 mm, and both sides of the plastic tube were sealed with solid glue. Thermoacoustic imaging was performed by placing plastic tubes equipped with the three components side by side at the same level. In addition, we further explored the TAI of *ex vivo* porcine blood at 1 h, 2 h, 3 h, 4.5 h and 6 h by placing it into a plastic thin-walled tubes with an internal diameter of 7 mm.

### Establishment of a canine intracerebral hemorrhage model and detection of canine intracerebral hemorrhage by microwave thermoacoustic imaging

Healthy beagles (*n* = 5, male; weight: 5–7 kg) used in the experiment were obtained from the Sichuan Institute of Musk Deer Breeding. All dogs were fasted the night before the experiment. The intracerebral hemorrhage model was established by canine autologous blood injection. The process of the intracerebral hemorrhage model was as follows: A beagle was anesthetized by intraperitoneal injection of 3% pentobarbital sodium at a dose of 1 ml/kg (body weight) + 2 ml. The dog was fixed on the operating table in a prone position, and a clean, thick towel was used to keep it warm. The head skin was sterilized after hair removal with a pet shaving device. A scalpel was used to cut the scalp in a 1 cm circle along the upper margin of the canine superciliary arch to the front of both ears, separated the temporalis muscle layer by layer, and scraped the periosteum. A craniotomy drill was used to drill a hole 5 mm from the left or right side of the midline of the top of the head (3 left side and 2 right side). During the drilling process, attention was given to gentle movements, and the wound was washed with 0.9% normal saline to reduce the temperature of the grinding area. The whole grinding time was approximately 50 min. During the operation and experiment, the state of the dog was noted at all times. If the dog trembled, 0.1 ml sumianxin was injected into the abdominal cavity for auxiliary anesthesia. After successful drilling, once the dog was fully hemostatic, the skin was sutured. The skulls of all dogs were intact at the time of TAI imaging, except for the small cranial boreholes.

Before the intracerebral hemorrhage model was made in each dog, ultrasound examination and TAI imaging were performed, and then blood injection was carried out to generate the intracerebral hemorrhage model. Five milliliters of blood were taken from the femoral artery of the dog and placed in an anticoagulant tube. Two milliliters of blood were drawn with 5 ml syringes. The dura was punctured vertically, the needle was slowly inserted into the intracranial area (the area 1.5–2 cm away from the syringe needle was clamped with hemostatic forceps to ensure a controlled injection depth), and autologous blood was injected slowly. The vital signs of the dog were closely observed during blood injection. After injection, the needle remained in the brain for 5 min and then was removed slowly. TAI imaging and ultrasound examination were performed at 0.5 h, 1 h, 2 h, 3 h, 4 h, 4.5 h, 5 h and 6 h after modeling. The same transducer was used during the process of TAI and ultrasound examination, and the probe position was kept motionless during each TAI and ultrasound examination. After each ultrasound examination, the position of the probe interface was only switched to ensure that the images obtained by ultrasound and TAI examination were from the same plane. All dogs remained deeply anesthetized during the experiment. Finally, the dogs were sacrificed by air embolization under anesthesia, and the brain was removed and prepared for pathological examination.

## Results

### Comparison of thermoacoustic signals in deionized water, fresh *ex vivo* porcine blood and *ex vivo* porcine brains

**FIGURE 2:**
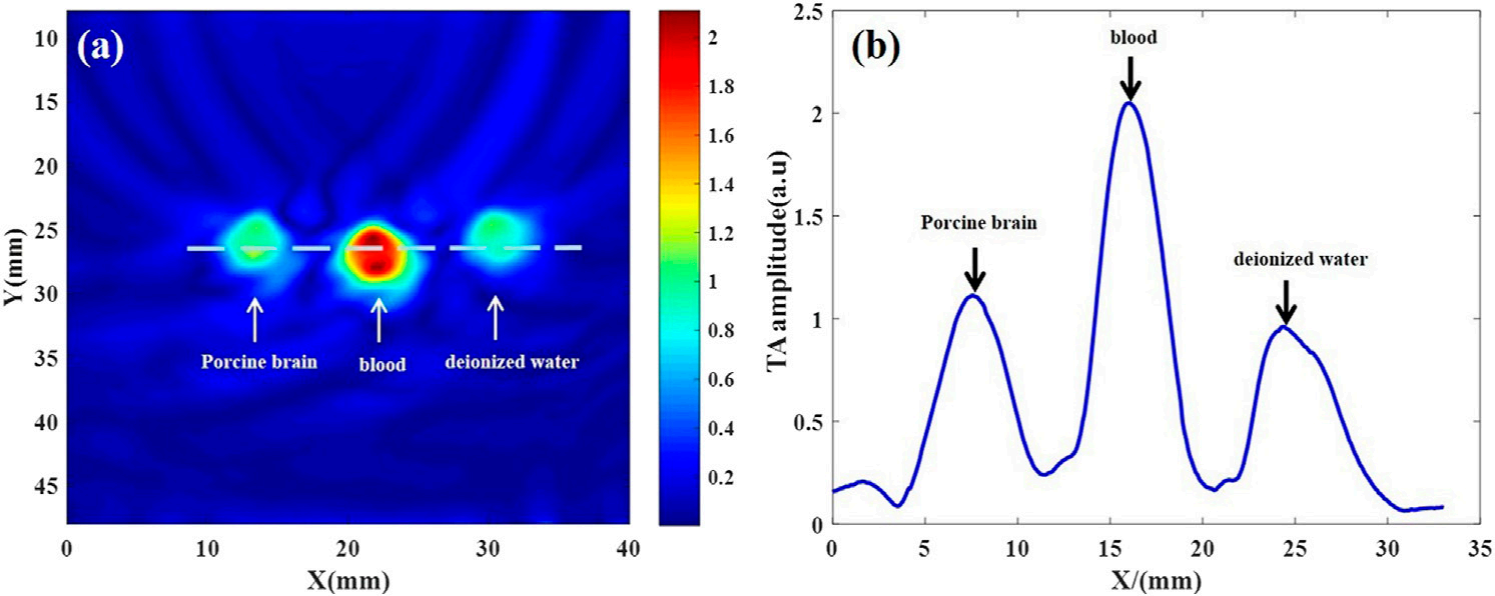
Thermoacoustic imaging of fresh *ex vivo* porcine brain, blood and deionized water. **(A)** Thermoacoustic image of *ex vivo* porcine brain, blood and deionized water. **(B)** The one-dimensional distribution curve of relative thermoacoustic signal amplitude along the white dashed line shown in **(A)**.

**FIGURE 3:**
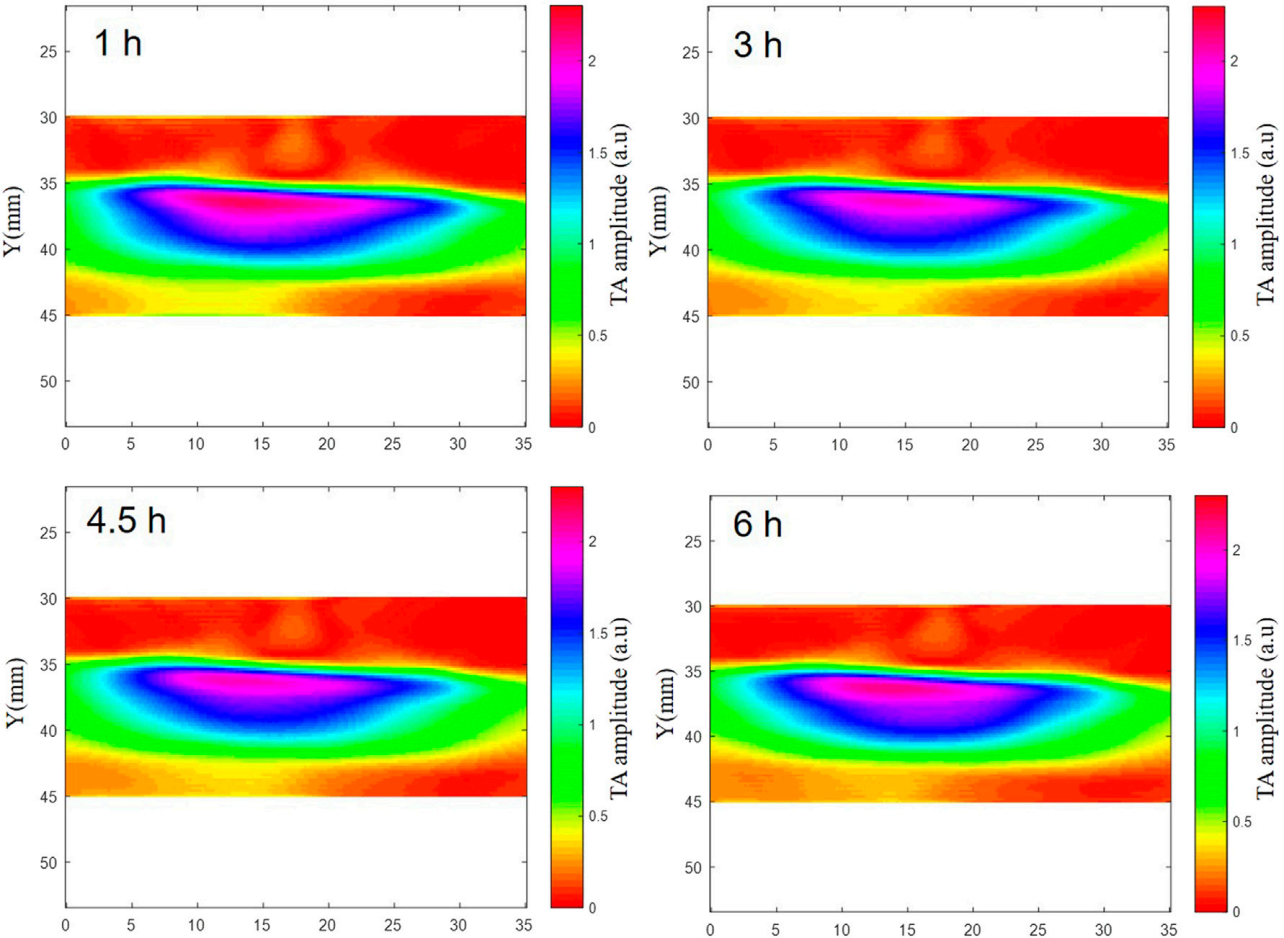
The TAI of fresh *ex vivo* porcine blood at 1 h, 2 h, 3 h, 4.5 h and 6 h. The results showed that there was no significant change in the thermoacoustic signals of fresh *ex vivo* porcine blood at 1 h, 2 h, 3 h, 4.5 h and 6 h.

TAI was performed on deionized water, isolated blood and isolated brain tissue simultaneously. The results showed that there were significant differences in microwave thermoacoustic signals among the three groups. The intensity of the thermoacoustic signal of blood was significantly higher than that of *ex vivo* porcine brain tissue and deionized water (Figure 2). In addition, there was no significant change in the thermoacoustic signals of fresh *ex vivo* porcine blood at 1 h, 2 h, 3 h, 4.5 h and 6 h (Figure 3).

### Thermoacoustic imaging and ultrasonography of canine intracerebral hemorrhage

**FIGURE 4:**
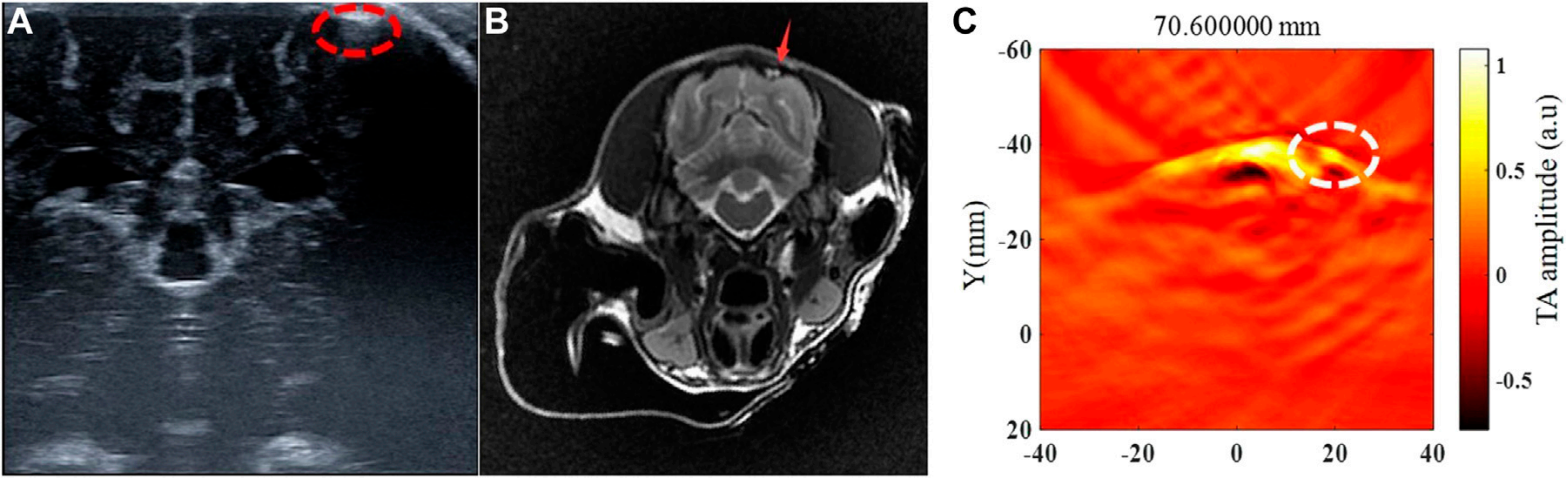
Ultrasonography, MRI and thermoacoustic imaging after 6 h of the canine intracerebral hemorrhage modeling. This is a case of canine intracerebral hemorrhage model with blood injected into cerebral sub-cortex. **(A)** Ultrasound showed the hemorrhage area as hyperechoic located at the left cerebral sub-cortex (red circle). **(B)** T2WI of MRI presented hemorrhage area as hyper-intensity located at the left cerebral sub-cortex (red arrow). **(C)** Thermoacoustic imaging showed a hyper-intensity of the hemorrhage area at the same location as presented at ultrasound and MRI (white circle).

**FIGURE 5:**
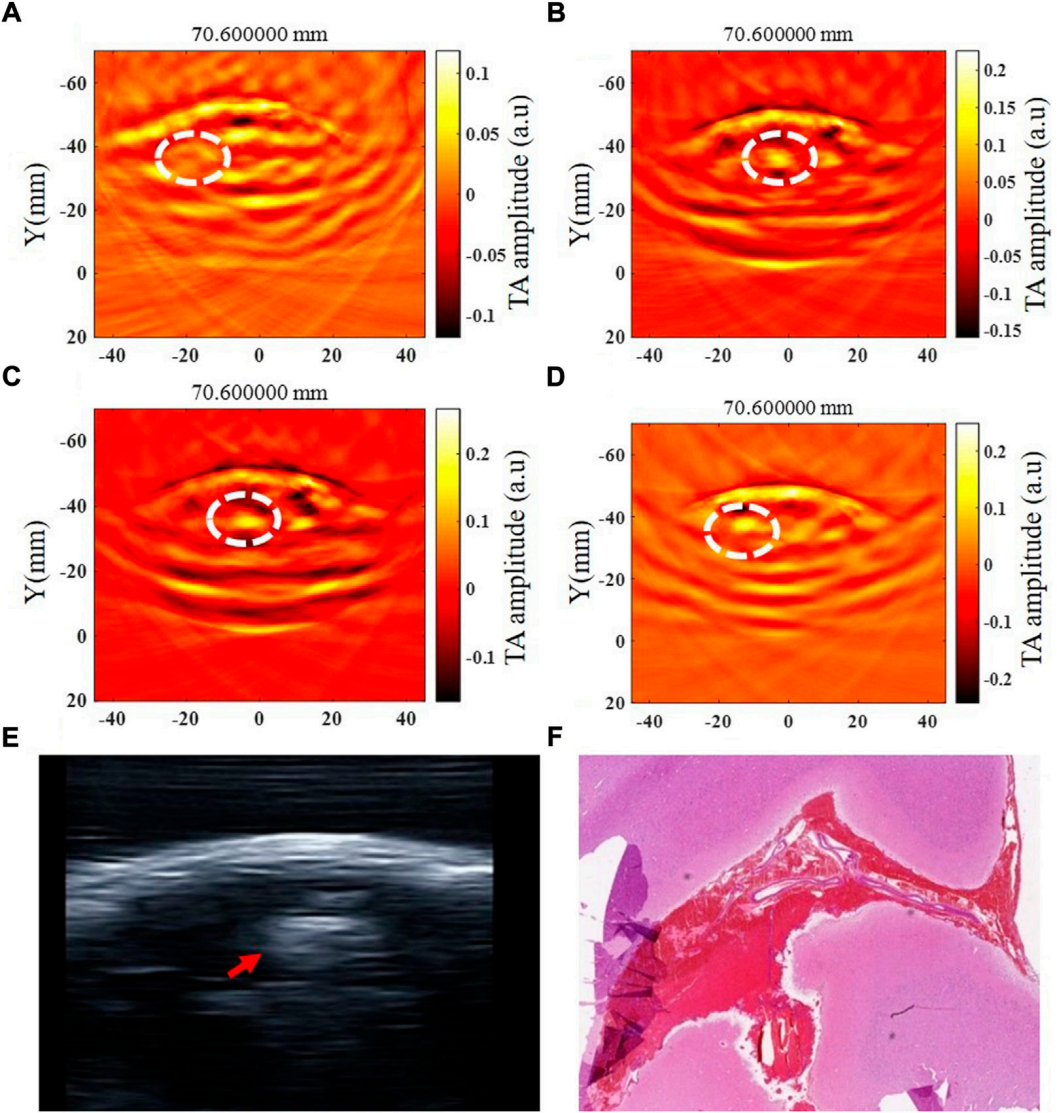
Thermoacoustic imaging, ultrasound and pathological examination of a same canine intracerebral hemorrhage. **(A–D)** TAI images at 0.5 h, 1 h, 3 h and 6 h after modeling, respectively (white circle shows the hemorrhagic area). **(E)** Ultrasound image of canine intracerebral hemorrhage (white circle shows the hemorrhagic area). **(F)** Pathological image of canine intracerebral hemorrhage.

The intracerebral hemorrhage model of five beagles was successfully established, and no dogs died during the experiment. The blood was injected in the right brain parenchyma in two beagles and in the left brain parenchyma in the other three beagles. The injection depth was 1.5 cm–2 cm within the brain parenchyma in four of five beagles, and the other one was injected into the cerebral subcortex due to a thicker scalp. The beagle with the thicker scalp underwent further MRI examination after 6 h of modeling (Figure 4). The time of data collection of TAI for each examination of the canine brain was 1 minute. At 0.5 h, 1 h, 2 h, 3 h, 4 h, 4.5 h, 5 h and 6 h after modeling, TAI and ultrasonography can be used to image intracerebral hemorrhage in the dogs. The hematomas presented hyperintensity in TAI. By comparing ultrasonography and pathology, the size and location of intracerebral hemorrhage in TAI were in line with those of the other groups (Figure 5).

## Discussion

In this study, we at first compared the thermoacoustic signal (TAS) in deionized water, fresh *ex vivo* porcine blood and *ex vivo* porcine brain, and the results showed that the intensity of the TAS of blood was significantly higher than that of brain tissue and deionized water, which is related to the apparent difference in conductivity between blood and brain tissues (Andreuccetti et al., 2021). These results provide a solid theoretical basis for further study on thermoacoustic imaging (TAI) in the detection of intracerebral hemorrhage in living animals. Moreover, we demonstrated, for the first time, the ability of TAI to detect intracerebral hemorrhage in large animals by establishing a canine hemorrhagic stroke model with ultrasound and pathological findings as a reference diagnosis.

In previous studies, TAI has demonstrated its potential for brain imaging in experiments on *ex vivo* (Yuan and Wang, 2006; Yan et al., 2019) and phantom (Huang et al., 2017) brain tissues. However, the dielectric properties of brain tissue may change due to a long time *in vitro* or preservation in formalin or mineral oil (Yuan and Wang, 2006), and the experimental results may not necessarily reflect the real status of brain imaging *in vivo*. Similarly, a phantom cannot simulate the complex electromagnetic environment of real living brain tissue. Thus, it is insufficient to prove that TAI is applicable to living tissues differently. Recently, studies (Zhao et al., 2017; Zhao et al., 2020) used TAI for brain imaging in living rats and demonstrated the feasibility of TAI in detecting germinal matrix hemorrhage in neonatal mice *in vivo*. These studies provide good evidence for the application of TAI in brain imaging of living animals. However, the brain structure of humans and rodents has significant discrepancies, and the canine brain is more similar to that of humans (Johnson et al., 20212020). Therefore, we explored the application of TAI in a canine intracerebral hemorrhage model in this study. TAI was performed by injecting blood into the brain parenchyma at different locations in beagles in the study, and the results showed that TAI could visualize intracranial hematomas in all cases. However, the depth of the hematomas in this study was only within 2 cm. Due to the small brain size of dogs, the cerebral cortex below 2 cm reached the lateral ventricle, so the experiment did not explore the modeling of hematoma in the deeper part of the brain, which is a limitation of this study. Of course, the detection of hematoma in the deeper part still needs further study.

In this study, we used a pulsed 3.0 GHz microwave source to transmit microwaves, which can penetrate several centimeters into tissue, for TAI. Thus, the penetration depth and spatial resolution of TAI for brain imaging are determined by the detected thermoacoustic signal. It is well known that skull-induced acoustic attenuation and scattering are the primary factors affecting ultrasound imaging of intracerebral hemorrhage, which also restricts the penetration depth and spatial resolution of TAI in this research. However, for TAI with a pulse width of approximately 600 ns in our experiment, the frequency of the thermoacoustic induced ultrasonic signal is approximately 1 MHz, which can penetrate the dog’s skull well and can provide mm-scale spatial resolution. In sum, this study demonstrated the feasibility of TAI in detecting hemorrhagic stroke *in vivo*. While the results obtained are encouraging, improvements are still needed before we can use this type of imaging for human brains. For example, several studies (Venkatasubramanian et al., 2011; Chen et al., 2021) have shown that the brain tissue around hematoma after hemorrhagic stroke will gradually develop edema and peak at 3–4 days, while the main purpose of this study is to explore the detection of intracerebral hemorrhage by TAI in the early stage. Therefore, whether perihematomal edema and the condition of the tissue 6 h after hemorrhage affect the detection of TAI remains to be further studied.

## Conclusion

In conclusion, this is the first experimental study to explore the use of TAI in the detection of intracerebral hemorrhage in large live animals (canine). The results indicated that TAI could be used to detect canine intracerebral hemorrhage and has the potential to be a rapid and noninvasive method for the detection of intracerebral hemorrhage in humans. In addition, with the miniaturization of microwave sources and rapid imaging speed, TAI is expected to be used in the future for pre-hospital detection of human intracerebral hemorrhage.

## Data Availability

The raw data supporting the conclusion of this article will be made available by the authors, without undue reservation.
